# Characterization of neutralizing antibodies reacting with the 213-224 amino-acid segment of human galectin-9

**DOI:** 10.1371/journal.pone.0202512

**Published:** 2018-09-11

**Authors:** Claire Lhuillier, Clément Barjon, Valentin Baloche, Toshiro Niki, Aurore Gelin, Rami Mustapha, Laetitia Claër, Sylviane Hoos, Yoichi Chiba, Masaki Ueno, Mitsuomi Hirashima, Ming Wei, Olivier Morales, Bertrand Raynal, Nadira Delhem, Olivier Dellis, Pierre Busson

**Affiliations:** 1 CNRS, UMR 8126, Villejuif, France; 2 Gustave Roussy, Université Paris-Saclay, Villejuif, France; 3 Univ Paris Sud, Université Paris-Saclay, Le Kremlin-Bicêtre, France; 4 Cellvax, Romainville, France; 5 Department of Immunology, Faculty of Medicine, Kagawa University, Takamatsu, Kagawa, Japan; 6 GalPharma Co., Ltd., Takamatsu, Kagawa, Japan; 7 CNRS, UMR 8161, IRCV group, Institut de Biologie de Lille, Lille, France; 8 H-Immune Therapeutics, Paris, France; 9 Plate-forme de Biophysique Moléculaire, Institut Pasteur, Paris, France; 10 Department of Pathology and Host Defense, Faculty of Medicine, Kagawa University, Takamatsu, Kagawa, Japan; 11 Department of Gastroenterology & Neurology, Faculty of Medicine, Kagawa University, Takamatsu, Kagawa, Japan; 12 INSERM, UMR-S 1174, Univ Paris Sud, Université Paris-Saclay, Orsay, France; University of British Columbia, CANADA

## Abstract

Extra-cellular galectin-9 (gal-9) is an immuno-modulatory protein with predominant immunosuppressive effects. Inappropriate production of gal-9 has been reported in several human malignancies and viral diseases like nasopharyngeal, pancreatic and renal carcinomas, metastatic melanomas and chronic active viral hepatitis. Therefore therapeutic antibodies neutralizing extra-cellular gal-9 are expected to contribute to immune restoration in these pathological conditions. Two novel monoclonal antibodies targeting gal-9 –Gal-Nab 1 and 2—have been produced and characterized in this study. We report a protective effect of Gal-Nab1 and Gal-Nab2 on the apoptotic cell death induced by gal-9 in primary T cells. In addition, they inhibit late phenotypic changes observed in peripheral T cells that survive gal-9-induced apoptosis. Gal-Nab1 and Gal-Nab2 bind nearly identical, overlapping linear epitopes contained in the 213–224 amino-acid segments of gal-9. Nevertheless, they have some distinct functional characteristics suggesting that their three-dimensional epitopes are distinct. These differences are best demonstrated when gal-9 is applied on Jurkat cells where Gal-Nab1 is less efficient than Gal-Nab2 in the prevention of apoptotic cell death. In addition, Gal-Nab1 stimulates non-lethal phosphatidylserine translocation at the plasma membrane and calcium mobilization triggered by gal-9 in these cells. Both Gal-Nab1 and 2 cross-react with murine gal-9. They bind its natural as well as its recombinant form. This cross-species recognition will be an advantage for their assessment in pre-clinical tumor models.

## Introduction

Galectins constitute a family of animal proteins defined by their binding specificity for glycans containing a β1–3 or β1–4 galactosyl bond carried either by glycoproteins or glycolipids. The domains of galectins that directly interact with carbohydrate ligands are called CRDs (for “carbohydrate recognition domains”) [1, 2]. The CRDs are made of about 135 amino acids (aa) forming a groove in which the carbohydrate ligand can bind. Interaction with a galactosyl bond is crucial for binding of each CRD to its physiological ligands. However, the binding specificity of each type of galectin is further specified by the atoms and molecules located at the periphery of the galactosyl bond which also interact with the CRDs.

Galectin-9 (gal-9) belongs to the category of “tandem-repeat” galectins containing two CRDs with distinct specificity linked by a flexible peptide chain called “linker peptide” (three other human “tandem-repeat” galectins are galectin-4, -8 and -12). As a result of alternative splicing, gal-9 exists under three main isoforms characterized by the length of the linker peptide: long (49 aa), medium (27 aa) and short (15 aa,) abbreviated as gal-9L, gal-9M (also called Δ5) and gal-9S (also called Δ5/ Δ6)[3]. We do not yet know the functional differences between these isoforms although we know that the length of the linker peptide influences the relative mobility of the two CRDs [3].

In basal physiological conditions, gal-9 is weakly expressed in most tissues (with the greatest abundance in the thymus and kidney). Its expression increases in many cell types–including endothelial and epithelial cells—under the influence of the cytokines of the Th1 immune response especially interferon-γ (IFN-γ) [4, 5]. Gal-9 is trafficking in various cell compartments either as a soluble protein or bound to the cell membrane network. It is consistently found in the cytoplasm. Depending on the cell type, it is also detected in the nucleus and at the surface of the plasma membrane [6, 7]. Like other galectins, gal-9 has no signal sequence. However, it can be secreted by non-conventional pathways, either bound to nanovesicles called exosomes, or under a soluble form by mechanisms which are not yet fully understood [6, 8, 9]. Distinct functions have been assigned to intracellular, cell surface and extracellular gal-9 [3]. Both intracellular and cell surface gal-9 have an impact on cell signaling and contribute to the organization of cell polarity. Cell surface gal-9 plays a role in contacts with neighboring cells and adhesion with extracellular matrix. When released in the extracellular medium, gal-9 acts like a cytokine with multiple immune-modulatory—mainly immuno-suppressive—activities involving several target cells. It promotes the expansion of regulatory T cells (Tregs) and strengthens their immunosuppressive activity, while it reduces the development of Th17 cells [10–16]. Gal-9 has been shown to induce apoptosis of CD4^+^ Th1 cells and CD8^+^ cytotoxic cells [17, 18]. Interestingly, it has also been implicated in the expansion of granulocytic myeloid-derived suppressor cells (MDSCs; CD11b^+^Ly-6G^+^F4/80^low^Ly-6C^low^) and more recently, in the promotion of Th2 /M2 differentiation that favors tumor progression in melanoma patients [19]. Our group and others have shown that gal-9 has a bi-phasic impact on peripheral T cells with early apoptosis in a majority of target cells and late phenotypic changes in surviving cells [20, 21]. It is still unclear what cell surface receptors of extracellular gal-9 are involved in the various effects mentioned above. Tim-3 was initially identified as one receptor of gal-9 on T cells [17, 22]. This has been confirmed in later studies. However, gal-9 can also bind other receptors such as CD44, dectin 1 or the protein disulfide isomerase [10, 23, 24].

There is mounting evidence that massive and inappropriate production of gal-9 has deleterious immunosuppressive effects in a number of viral and/or malignant diseases. The contribution of gal-9 to tumor or viral immune evasion was first reported by our group in publications dealing with Epstein-Barr virus-related nasopharyngeal carcinomas [8, 25]. Later on, similar observations were reported in chronic active viral hepatitis with or without hepatocellular carcinomas [26–28]. More recently, unwanted effects of gal-9 have been demonstrated or strongly suspected in a series of human malignancies without known viral etiology, including pancreatic and renal carcinomas, metastatic melanomas, a subgroup of lung carcinomas, glioblastomas and several types of acute myeloblastic leukemias [19, 22, 24, 29–31]. We believe that therapeutic antibodies neutralizing gal-9 are likely to become important therapeutic tools for the treatment of these diseases. Our general objective is to produce and characterize such antibodies. We have noticed that antibodies neutralizing extra-cellular gal-9 used in previous publications have not been precisely described in terms of affinities, sequences of variable segments, epitopes and cross-reactivity [22, 25]. Therefore, the aim of this report is to make these data publicly available for two novel neutralizing mAbs—Gal-Nab1 and Gal-Nab2—which are also well characterized in terms of functional activities. They were obtained following mouse immunization with the recombinant c-terminal part of gal-9. Their impact on the immuno-modulatory functions of extracellular gal-9 such as apoptosis induction, calcium mobilization or late phenotypic changes, was investigated *in vitro* using various functional assays based on Jurkat leukemic T cells or peripheral blood mononuclear cells (PBMCs). Intriguing functional differences were found between Gal-Nab1 and Gal-Nab2 although they bind to nearly identical linear epitopes (aa 213–224). These differences might be related to distinct contributions of other parts of the protein to the epitope/paratope interface. On the other hand, we found that both Gal-Nab1 and 2 react with murine recombinant and native gal-9 in two distinct types of binding assays. These characteristics might be helpful to use them in pre-clinical investigations.

## Materials and methods

### Reagents

The various forms of recombinant gal-9—human S and M isoforms and murine M isoform–were produced in Escherichia coli (E. coli) as GST fusion proteins. Tag-free proteins were purified by affinity chromatography on lactose-agarose column [32]. The c-terminus galectin-9 (residues 191 to 355 of the gal-9 long isoform) was produced in E. coli as a GST-fusion protein. The tag-free protein was purified by exclusion chromatography [7]. Only tag-free gal-9 proteins were used in our experiments. Lactose (β-lactose) was purchased from Sigma-Aldrich. In house anti-gal-9 mAbs (Gal-Nab1 and Gal-Nab2) were produced in mice as described in the next paragraphs and were both of the IgG1, κ isotype. The mouse control isotype antibody (control IgG) was from BioLegend. Commercial monoclonal anti-gal-9 antibodies were also used: 9M1-3 (BioLegend) and RG9-35 (BioLegend) directed against human and murine gal-9, respectively. A goat polyclonal antibody was used for detection of murine gal-9 by Western blotting (R&D System/Bio-Techne).

### Animal care and immunizations

Procedures used for mouse handling and immunization were reviewed and approved by the following Ethics committee: “comEth Afssa/ENVA/UPEC” sponsored by “Ecole Nationale Vétérinaire d’Alfort” and “Université Paris-Est- Créteil -Val de Marne” (decision # 14/02/12-1, February 14, 2012). BALB/c female mice age 6–8 weeks were purchased from Charles Rivers Laboratories (Saint Germain–Nuelles, France) and housed in pathogen-free conditions in filter cap cages holding a maximum of 5 animals with irradiated aspen chip bedding and cotton fiber nesting material. They were maintained on a 12/12 light/dark cycle, with *ad libitum* UV-treated water and RM1 rodent diet. Immunogenic mixtures were injected intra-peritoneally. Intermediate blood collections were done by puncture of the orbital sinus (50 μl with intervals of at least 15 days). The animals were monitored every day for signs of pain, such as immobility or restlessness, reduction of drinking and food intake. The persistence of abnormal behaviors for more than one day led to the euthanasia of animals with suffering presumption. Prior to spleen collection, mice were sacrificed by cervical elongation. Otherwise, mice were euthanatized by carbon dioxide asphyxiation.

### Production of anti-galectin-9 monoclonal antibodies

Immunizations were conducted at PX’Therapeutics (Grenoble, France) [7]. Five BALB/c female mice (eight weeks old) were immunized with the recombinant c-terminus galectin-9. Immunizations (40 μg of protein) were administered intraperitoneally at days 0, 22, 37 and 54 with complete Freund’s adjuvant for the first immunization, then with incomplete Freund’s adjuvant for subsequent injections.

Anti-gal-9 antibodies were detected in mouse sera and hybridoma supermatants, by ELISA (enzyme-linked immunosorbent assay). Briefly, microtiter plates were coated with 0.05 M carbonate/bicarbonate buffer pH 9.6, containing 50 ng human c-terminus gal-9 per well, during 1h at room temperature. After washing with phosphate buffered saline (PBS) containing 0.1% Tween-20, the wells were saturated with 3% bovine serum albumine (BSA) in PBS at room temperature for 1h. They were then incubated with mouse sera or raw hybridoma supernatants in PBS with 1% BSA at room temperature for 2h. After a washing step with PBS 0.1% Tween-20, the anti-galectin-9 antibody level was determined using horseradish peroxidase-coupled (HRP) goat antibodies to mouse IgG (Sigma-Aldrich, St Quentin Fallavier, France).

An immune response against the c-terminal part of gal-9 was confirmed by ELISA in the five immunized mice. Splenocytes were collected from the two best responding mice which were sacrificed three days after the last boost. These splenocytes were used in liquid or semi-solid fusions with Sp2/0 cells at a ratio of 5:1 and 2:1, respectively. Hybridomas supernatants were assessed using gal-9 ELISA. The semi-solid fusion was successful and a collection of monoclonal hybridomas secreting anti-gal-9 antibodies was obtained. For subsequent experiments, antibodies were purified from hybridoma supernatants using protein A affinity chromatography. The concentrations of endotoxins in the final solutions were checked using the following kit: ToxinSensor^TM^ Chromogenic LAL Endotoxin Assay (GenScript). They were below 3 EU/ml.

### Preparation and culture of galectin-9 target cells

The human T cell line Jurkat E6.1 (American Type Culture Collection) was grown in RPMI 1640 medium (Gibco-Life Technologies) supplemented with 10% fetal calf serum (FCS) at 37°C in a 5% CO_2_ humidified atmosphere. Human peripheral blood mononuclear cells (PBMCs) were obtained from anonymous healthy blood donors using standard density gradient centrifugation (Lymphocyte separation medium, Eurobio AbCys) and used for subsequent T cell purification or cultured at 37°C with 5% CO_2_ in RPMI 1640 medium supplemented with 10% FCS. CD3^+^ T cells were purified using magnetic separation of untouched CD3^+^ cells (pan T cell isolation kit; Miltenyi Biotec). For T cell activation, PBMCs or purified CD3^+^ cells were cultured in anti-CD3-coated plates in the presence of soluble anti-CD28 antibodies (0.5 μg/mL; Miltenyi Biotec).

### Apoptosis assessment

Jurkat cells were exposed in serum-free medium (Hybridoma-SFM, Gibco) to crude gal-9 or gal-9 previously pre-incubated for 30 min at 37°C with lactose, a control isotype antibody or anti-gal-9 mAbs at 2X concentrations. During the subsequent incubation with target cells, reagents were at 1X concentration, as indicated in the figure legends. After 24h of incubation, Jurkat cells were washed and stained with annexin-V (Thermo Fischer Scientific) and propidium iodide (PI; Sigma-Aldrich) before acquisition on the Accuri^®^ C6 flow cytometer (BD Biosciences). For assessment of primary T cells apoptosis, purified CD3^+^ cells were incubated in complete RPMI medium (10% FCS) and treated as indicated above. After 36h of incubation, CD3^+^ cells were washed, stained with annexin-V and PI and analyzed by flow cytometry.

### Measurement of cytosolic calcium concentration ([Ca^2+^]_cyt_)

[Ca^2+^]_cyt_ was recorded in Jurkat cells by a fluorimetric ratio technique [33]. The fluorescent indicator Indo-1 (4 μM; Invitrogen/Molecular Probes) was loaded by incubating the cells at room temperature under gentle agitation. Cells were then resuspended in Hepes Buffer Saline medium and treated with gal-9 (30 nM) preincubated or not with lactose (5 mM) or with anti-gal-9 mAbs (67 nM, i.e. 10 μg/mL). One million cells was put in a 1 cm width– 3 mL quartz cuvette, and inserted in a spectrofluorimeter (Varian Cary Eclipse), equipped with a thermostated cuvette holder. Excitation of Indo-1 was done at 360 nm, and emissions at 405 and 480 nm were recorded. Background and autofluorescence were subtracted from the values measured at 405 and 480 nm. Intracellular Ca^2+^ concentrations were calculated following the method already described by O. Dellis and collaborators [33]. Traces were given without SEM for clarity (SEM values were usually < 40 nM).

### Phenotypic analysis and cell sorting

After one week of culture with indicated treatments, PBMCs were analyzed for their central memory, effector memory or naive T cell phenotype, by incubation with PE-conjugated anti-CD45RO, FITC-conjugated anti-CCR7, and APC-conjugated anti-CD3 antibodies (Biolegend). Isotype-matched non-specific antibodies were used as negative controls. Staining was analyzed on viable cells on forward scatter/sideward scatter plot, and on the CD3^+^ population gate.

### Intracellular cytokine staining

After one week of culture with indicated treatments, PBMCs were restimulated with 50 ng/mL phorbol 12-myristate 13-acetate (PMA) and 500 ng/mL ionomycin (Santa Cruz biotechnolgy) in the presence of 10 μg/mL brefeldin A (Sigma-Aldrich) for 4h. Cells were washed and stained with APC-conjugated anti-CD3 antibody. Then, cells were fixed, permeabilized and stained with FITC conjugated anti-IL-2 and PE-conjugated anti-IFN-γ antibodies (Biolegend).

### Investigations of Gal-Nab1 and Gal-Nab2 binding to full length gal-9M by surface plasmon resonance (Biacore)

Assessment of antibody interactions with gal-9 was done by surface plasmon resonance using a T200 Biacore (GE Healthcare) device. In a first series of experiments, gal-9M was used as a ligand and covalently bound to the chip (Series S Sensor chip CM5, GE Healthcare) activated by NHS/EDC (mix of N-hydroxysuccinimide and 1-ethyl-3-(3-dimethyl-amino-propyl) carbodiimide**).** Covalent binding was done by injection of recombinant gal-9M in the microfluidic system at 10 μg/mL, at pH5, in acetate buffer. Purified Gal-Nab1 and Gal-Nab2 were used as the analytes. They were injected in the microfluidic system at various concentrations from 1.5 to 400 nM, with a flux of 40 μL/min, for 400s, in the presence of albumin (0.1 mg/mL) and tween 0.05%. The dissociation of the complex was followed for 500s. Regeneration of the chip was done with glycine (10 mM, pH 1.5).

Reciprocally, in the next series of experiments, Gal-Nab1 and Gal-Nab2 mAbs were used as ligands covalently bound to the CM5 chip activated by NHS/EDC. Covalent binding was done by injection of the purified antibodies in the microfluidic system at 10 μg/mL in acetate buffer, at optimal pH (5 for Gal-Nab1 and 4.5 for Gal-Nab2). The injection lasted 400s and was followed by a 900 s washing step. Recombinant gal-9M was used as the analyte. It was injected at various concentrations from 0.39 to 100 nM, with a flux of 40 μL/min, for 400s, in PBS with albumin, 0.1 mg/mL, and tween 0.05%. The dissociation of the complex was followed for 600s. Regeneration of the chip was done with water containing SDS 0.1% for 1 min twice.

Binding parameters, especially the K_D_ (dissociation constant) were calculated using the Biacore T200 evaluation software. The on-rate constant (K_on_ in s^-1^ M^-1^) was calculated from the ascending portion of the curve depicting the loading of the free Gal-Nab1 and 2 antibodies (analytes) on the immobilized gal-9-M (ligand) or the loading of the recombinant gal-9M (analyte) on the immobilized Gal-Nab1 and Gal-Nab-2 antibodies (ligands). The K_on_ characterizes how quickly the antibodies bind to their target. The off-rate constant (Koff in s^-1^ M^-1^) was calculated from the down-side of the curve depicting the elution of the analytes from the immobilized ligands. It characterizes how quickly the antibodies dissociate from their target.

### Investigations of Gal-Nab1 and Gal-Nab2 binding to a biotinylated peptide by surface plasmon resonance (Biacore)

In these experiments, the ligand was an oligopeptide called CTB containing the amino-acids 208 to 232 of gal-9L (176–200 and 164–188 of gal-9M and S, respectively), biotinylated at its c-terminus and bound to a streptavidin chip (Series S Sensor chip SA, GE Healthcare). In this 32-mer oligopeptide, the 25 gal-9 aa were flanked by stuffer aa not related to gal-9, designed to increase its solubility and accessibility (complete sequence with stuffer aa in italic: *KRE*FSTPAIPPMMYPHPAYPMPFITTIL*DRKK*(biotin)-OH). A biotinylated scramble oligopeptide was used as a control (complete sequence: biotin-PMTPYKPHTGK MYPIRQAPMAPFDSIERPMF-OH). To achieve streptavidin binding, the biotinylated oligopeptides were injected in the microfluidic system at a concentration of 500 ng/mL in PBS during at least 25s. Purified Gal-Nab1 and Gal-Nab2 mAbs were used as the analytes. They were injected at various concentrations from 0.05 to 3.2 nM, with a flux of 40 μL/min, for 700 s, in PBS with albumin 0.1 mg/mL and tween 0.05%. The dissociation of the complex was followed for 800s Regeneration of the chip was done with NaOH 500mM, SDS 0.1% and H_2_O. Binding parameters were calculated as previously explained for Biacore experiments on full length gal-9-M.

The same CTB and control peptides were used in a functional assay based on apoptosis protection of CD3^+^ T cells treated for 36h with gal-9 (gal-9S; 40 nM). Gal-Nab1, Gal-Nab2 and control IgG Abs (67 nM) were pre-incubated with the CTB or the scramble peptide (6.7 μM i.e. with a molar ratio of 100) for 30 min at 37°C prior to their pre-incubation with gal-9.

### Epitope mapping by oligopeptide competition

Epitope mapping for Gal-Nab1 and Gal-Nab2 mAbs was realized using 15 overlapping peptides of 15 aa (peptide 1 SAPGQMFSTPAIPPM to peptide 15 MMYPHPAYPMPFITT). These peptides were covering a segment of gal-9 mapping between aa 202 and 230 (using gal-9L coordinates; or 158 and 186 using gal-9S coordinates). This segment was chosen on the basis of prior experiments performed in our laboratory (data not shown). Gal-Nab1 and Gal-Nab2 were diluted at a concentration of 0.06 and 0.3 μg/mL, respectively (i.e. at the effective concentration 50 previously determined) and pre-incubated with increasing concentrations of each peptide (from 0.0001 to10 μg/mL). Then, mAbs/peptides mixtures were added to 96-wells plates coated with gal-9. Secondary HRP-conjugated anti-mouse antibodies were used to detect mAbs binding to gal-9. Experimental absorbance values (A_exp_) or background values of absorbance (A_0;_ measured in wells without mAbs/peptides) were subtracted from maximal absorbance values (A_max_; maximum binding of mAbs without peptides) and the percentages of inhibition were calculated as follows: [(A_max_—A_exp_) / (A_max_—A_0_)] *100. Results are shown only for the peptides 7 to 14 because the percentage of inhibition was very low with the other peptides (1 to 6 and 15).

### Determination of the hypervariable regions for Gal-Nab1 and Gal-Nab2

Total RNA was extracted from hybridoma cell pellets using a Trizol solution (Tri Reagent, Euromedex). It was then reverse-transcribed using the ImProm-II™ Reverse Transcription System from Promega. cDNA segments encoding the variable regions of the heavy and light chains of Gal-Nab1 and 2 were amplified using the Phusion “hot start” polymerase (Thermo Fisher Scientific) and flanking degenerated primers recommended by Srebe et al. [34]. Fragments amplified by efficient primers were excised from agarose gels and subjected to a second round of amplification using the same primers. The resulting fragments were ligated in the pJet1.2/blunt plasmid (Thermo Fisher Scientific), cloned in E. coli and subjected to Sanger sequencing. Hypervariable regions (CDR 1, 2 and 3 of heavy and light chains) were identified using the softwares provided by http://www.bioinf.org.uk/abs/.

### Determination of monoclonal antibody cross-reactivity with murine gal-9 by ELISA and cold immuno-precipitation

A comparative assessment of mAbs binding to human gal-9M and S, murine gal-9M and several human galectins (1–4, 7, 8 and 10) was done by direct ELISA. Briefly, Gal-Nab1, Gal-Nab2 or IgG1 control mAbs were coated in a 96-well plate. Then, biotinylated galectins were incubated at 1 nM and detected by the classic streptavidin-HRP method. For cold immuno-precipitation, proteins were extracted from a BALB/c mouse thymus using a lysis buffer containing 10 mM Tris HCl pH 7.4, 150 mM NaCl, 1% Triton, 0,05%SDS and protease inhibitors (Complete, Roche). Primary antibodies (Gal-Nab1, Gal-Nab2, 9M1-3, RG9-35) and control mouse IgG1 were loaded on magnetic beads bearing covalently bound anti-mouse IgG sheep antibodies (10 μg purified antibodies for 1.6 X 10^6^ beads) (M280 Dynabeads, Thermo Fischer Scientific). Immune complexes were formed by incubation of the thymic protein extract (120 μg proteins in 600 μl of lysis buffer) with antibody loaded beads (4 X 10^7^) for a time interval of 3h to 18h at 4°C with rotative agitation. Magnetic beads loaded with immune complexes were then washed 7 times with lysis buffer (without SDS and protease inhibitors). Finally, immune complexes were eluted by boiling the beads for 4min in Laemmli buffer and loaded on a PAGE gel for Western blot analysis.

### Statistics

Statistical analyses were performed by one-way ANOVA followed by Dunnet post-test using GraphPad Prism 7 software. p <0.05 was defined as a statistically significant difference. Where indicated * p <0.05; ** p<0.01; *** p<0.001; ns: not significant.

## Results

### Impact of anti-gal-9 antibodies on gal-9-induced cell death and calcium mobilization in the Jurkat T cell line

**Fig 1:**
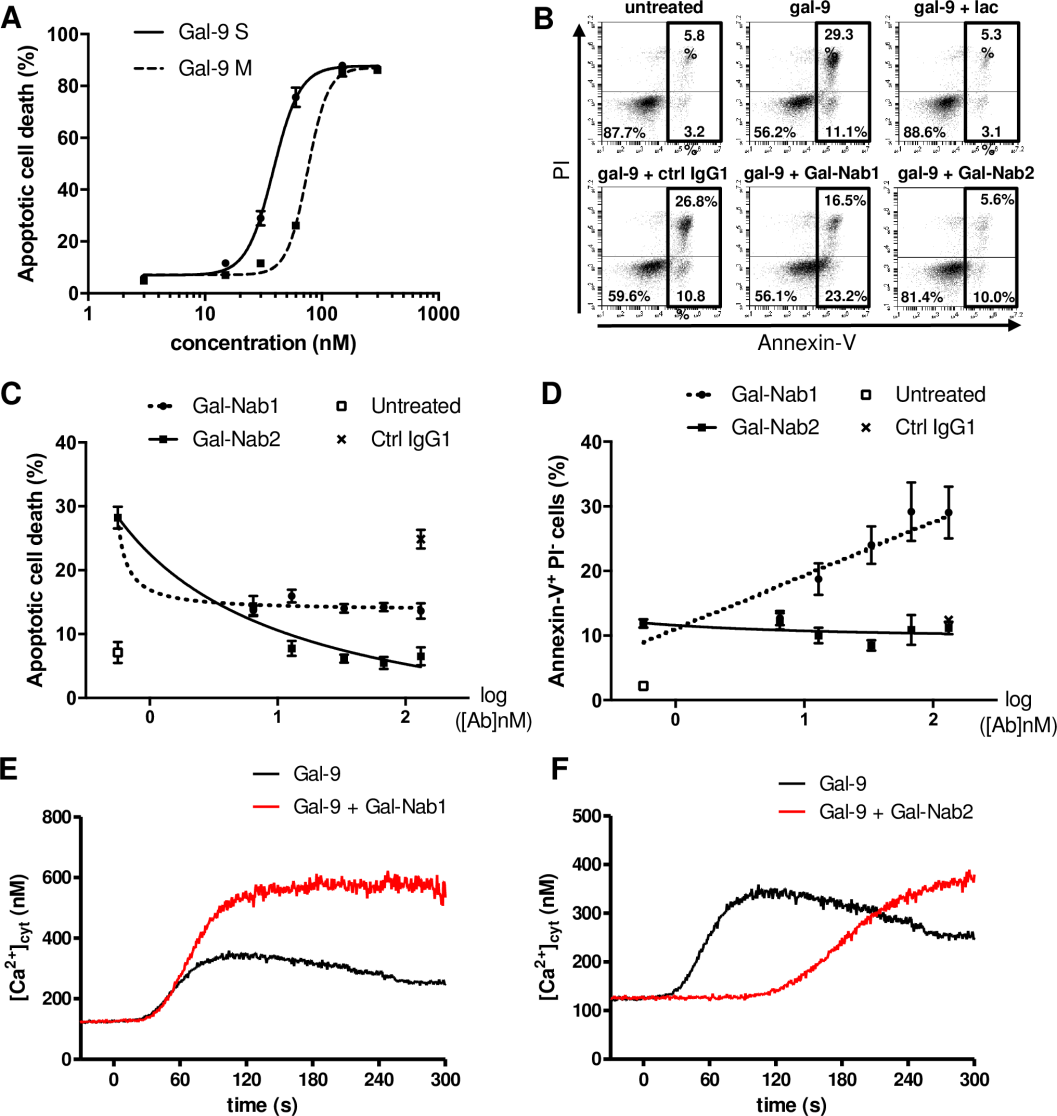
Jurkat cell responses to exogenous gal-9 in the presence of Gal-Nab1 and Gal-Nab2 antibodies. **A.** Jurkat cells were treated during 24h with increasing concentrations (3 to 300 nM) of human recombinant gal-9 (gal-9S or M) and percentages of apoptotic cell death were assessed by flow cytometry analysis of annexin-V^+^ PI^+^ cells (technical duplicates). **B.** Example of flow cytometry plots for Jurkat cells treated or not with gal-9 (gal-9S; 40 nM) alone or in combination with lactose (5 mM), control isotype mAbs (ctrl IgG1) or anti-gal-9 mAbs (Gal-Nab1 and Gal-Nab2) at 67 nM (i.e. 10 μg/mL) followed by annexin-V and PI staining after 24h. **C-D.** Dose-response curves for apoptotic cell death (annexin-V^+^ PI^+^) **(C)** or PS translocation (annexin-V^+^ PI^-^) **(D)** in Jurkat cells treated for 24h with increasing concentrations of Gal-Nab1 and Gal-Nab2 (6 to 130 nM). Empty squares indicate the percentages obtained in conditions without gal-9. Black crosses indicate the percentages obtained with isotype control IgG1 mAbs used at maximal concentration (130 nM). Data are presented as means ± SEM of four biological replicates. **E**-**F.** Variations of [Ca^2+^]_cyt_ in Jurkat cells treated with gal-9 (40 nM, added at t = 0 s) alone (black line) or with gal-9 combined with Gal-Nab1 **(E)** or Gal-Nab2 **(F)** (red line). [Ca^2+^]_cyt_ was assessed by Indo-1 fluorimetry as described under “Materials and Methods”. Data presented here are representative of three similar independent experiments.

A series of monoclonal antibodies reacting with gal-9 were obtained by mouse immunization with a recombinant protein representative of the c-terminal portion of gal-9L (long isoform) containing amino-acids 191 to 355 as previously described [7]. Two of them, named Gal-Nab1 and Gal-Nab2, were selected as potential neutralizing antibodies on the basis of preliminary experiments (both are of the IgG1-ϰ isotype). Their reactivity with gal-9 was identical in the presence as well as in the absence of lactose (S1 Fig). To confirm their neutralizing activity, we first used the human leukemic T cell line Jurkat, which is known to be responsive to extracellular gal-9 [35, 36]. In a previous report, we have shown that in serum-free conditions, Jurkat cells are sensitive to nanomolar concentrations of gal-9 [20]. We first compared the ability of two isoforms of gal-9 (gal-9S and gal-9M) to induce apoptotic cell death in Jurkat cells by assessing the percentages of annexin-V-positive and propidium iodide-positive (annexin-V^+^PI^+^) cells after 24h of treatment (Fig 1A). Percentages of apoptosis reached a plateau at about 85–90% with 130 and 250 nM of gal-9S and gal-9M, respectively. The mean effective concentrations inducing apoptotic death in 50% of the cells (EC50) were 40 and 80 nM for gal-9S and gal-9M, respectively. Based on these data, 40nM was chosen as the common working concentration used for gal-9S and M in all subsequent experiments. Apoptosis induction was chosen as one initial end-point to assess the neutralizing capacity of our mAbs. For correct interpretation of our following results, one needs to keep in mind that extra-cellular galectins often induce both apoptotic death in a fraction of their target cells (rapid succession of annexin-V and PI staining) and a process of non-lethal phosphatidylserine (PS) translocation in another fraction (annexin-V positivity in the absence of subsequent PI staining) [37]. In this regard, distinct effects were obtained when using Gal-Nab1 and Gal-Nab2. As shown in Fig 1B, Gal-Nab2 completely abrogated gal-9-induced apoptosis (annexin-V^+^PI^+^) in Jurkat cells, but did not prevent the non-lethal PS translocation (annexin-V^+^PI^-^). In contrast, Gal-Nab1 was less efficient in reducing the percentage of apoptotic cell death, and rather increased the fraction of annexin-V^+^/PI^-^ cells. As expected, both PS translocation and apoptotic cell death were not affected by the control IgG. This suggests that Gal-Nab1 favors the translocation of PS induced by gal-9 although it reduces its capacity to induce apoptosis. This functional difference between Gal-Nab1 and Gal-Nab2 was confirmed by the dose-response curves in Fig 1C and 1D where Gal-Nab2 decreased the percentage of apoptotic cells (to a level equivalent to the untreated condition) without modifying the unlethal PS translocation induced by gal-9. On the other hand, Gal-Nab1 reduced by about 50% the level of apoptosis cell death and even increased the percentage of annexinV^+^/PI^-^ cells.

Another known effect of gal-9 on Jurkat cells is the induction of calcium mobilization [35, 38]. We previously demonstrated that the rapid calcium mobilization induced by gal-9 in Jurkat cells–which occurs in less than 1 minute—is not required for apoptosis induction [20]. Thus, we wondered whether our mAbs could neutralize this additional independent effect triggered by extracellular gal-9. Using the free calcium probe Indo-1, intracytosolic calcium release was assessed in Jurkat cells treated with recombinant gal-9. Like for apoptosis assays, experiments were performed in serum-free medium using nanomolar concentrations of gal-9. Calcium release was rapid, occurring within 30s after gal-9 addition (Fig 1E and 1F). It was inhibited by pre-treatment with 50 μM 2-APB, a Store-Operated Calcium Entry inhibitor (data not shown) [33]. Calcium mobilization was delayed of about 90s after gal-9 pre-incubation with Gal-Nab2 (Fig 1F). In contrast, calcium mobilization was not delayed and its amplitude was even increased when gal-9 was pre-incubated with Gal-Nab1 (Fig 1E). This observation strengthens the idea that Gal-Nab1 and 2 have distinct effects on the interactions of gal-9 with Jurkat cells.

### Anti-gal-9 mAbs neutralize gal-9-induced apoptosis of primary T cells

**Fig 2:**
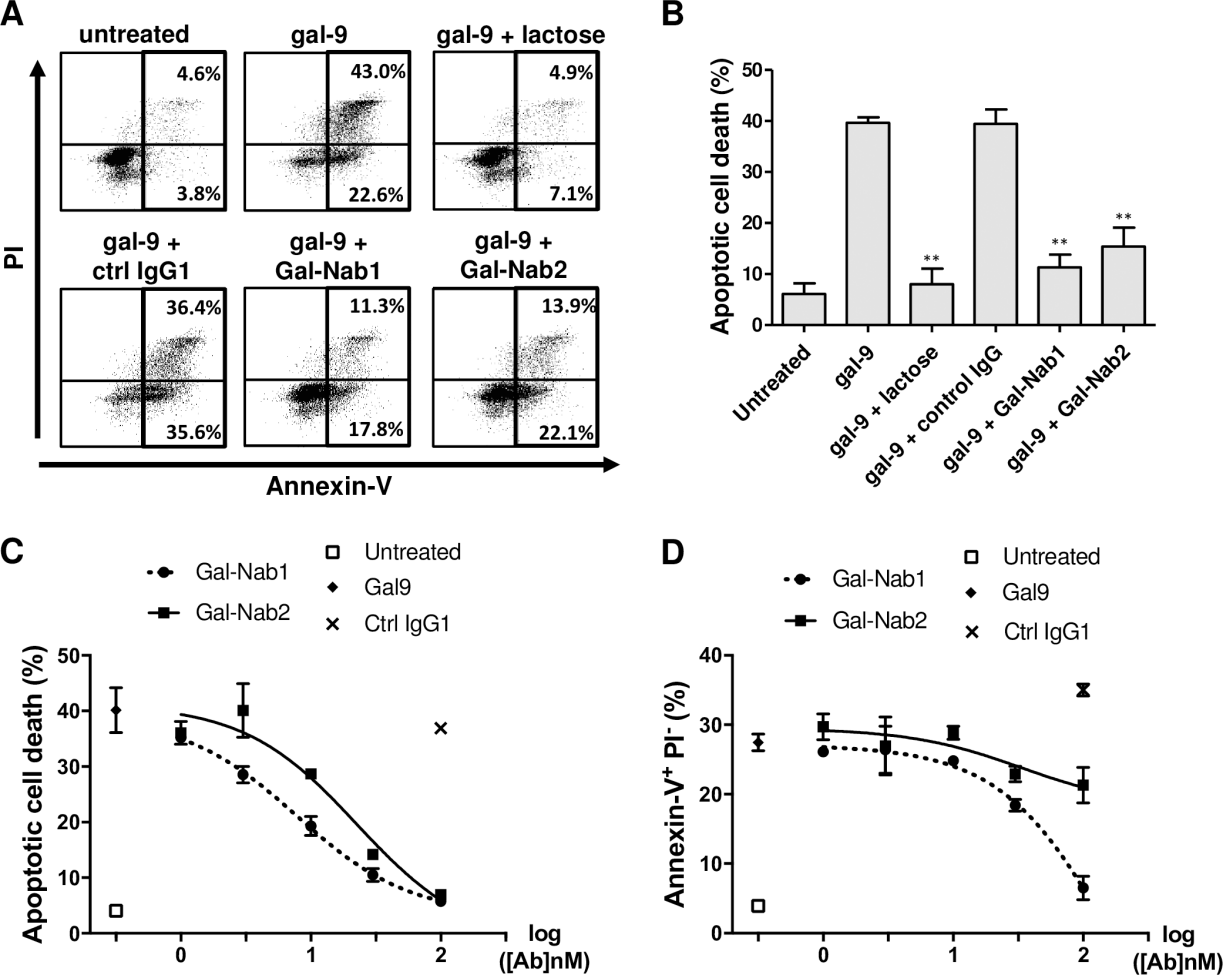
Anti-gal-9 mAbs efficiently neutralize gal-9-induced apoptosis in primary T cells. CD3^+^ T-cells were isolated from healthy donors, activated by a combination of CD3/CD28 antibodies and treated or not with gal-9 (gal-9S; 40 nM) alone or in combination with lactose (5 mM), control isotype mAbs (ctrl IgG1) or anti-gal-9 mAbs (Gal-Nab1 and Gal-Nab2) at 67 nM (i.e. 10 μg/mL). After 36 h, they were subjected to annexin-V/PI staining and flow cytometry analysis. **A.** Examples of flow cytometry plots for purified CD3^+^ cells from one donor. **B.** Synthesis of data from 3 similar experiments made with CD3^+^ cells from 3 donors. **C and D.** Dose-response curves for apoptotic cell death (annexin-V^+^ PI^+^) **(C)** or PS translocation (annexin-V^+^ PI^-^) **(D)** in activated CD3^+^ cells treated for 36h with gal-9 combined with increasing concentrations of Gal-Nab1 and Gal-Nab2 (0.3 to 100 nM). Empty squares indicate the percentages obtained in conditions without gal-9. Black crosses indicate the percentages obtained with isotype control IgG1 mAbs used at maximal concentration (100 nM). Data are presented as means ± SEM of three independent experiments made with three distinct donors.

Several studies have shown that gal-9 induces the apoptosis of peripheral primary T cells *in vitro* [17, 39]. Therefore, we investigated the capacity of our anti-gal-9 mAbs to protect primary T cells from apoptosis induced by exogenous gal-9. CD3^+^ cells from human healthy donors were activated by anti-CD3/CD28 Abs and treated with gal-9 for 36h in the presence or absence of Gal-Nab1, Gal-Nab2 or control antibodies. Gal-9 induced non-lethal PS translocation (annexin-V^+^PI^-^) and apoptotic cell death (annexin-V^+^PI^+^) in CD3^+^ cells (Fig 2A and 2B). Both PS translocation and apoptotic cell death were almost entirely reversed by addition of lactose (5 mM) (Fig 2A and 2B). Apoptotic cell death was dramatically reduced (about 75–80% reduction) by both Gal-Nab1 and Gal-Nab2 (Fig 2A and 2B). The impact on PS translocation was much weaker; Gal-Nab2 had almost no effect (Fig 2A and 2B). These observations were confirmed when CD3^+^ cells were treated with gal-9 in combination with increasing concentrations of the antibodies (dose/response curves; Fig 2C and 2D). Gal-Nab1 was slightly more efficient than Gal-Nab2 to prevent apoptotic death of primary T cells (EC50 of 10 and 25 nM, respectively). Higher concentrations of Gal-Nab1 were required to reduce non-lethal PS translocation (EC50 of 50 nM) whereas Gal-Nab2 had almost no effect on this alteration.

### Anti-gal-9 mAbs inhibit the late emergence of Th1-like and T_CMs_-like T cells observed in PBMCs surviving apoptosis induced by gal-9

**Fig 3:**
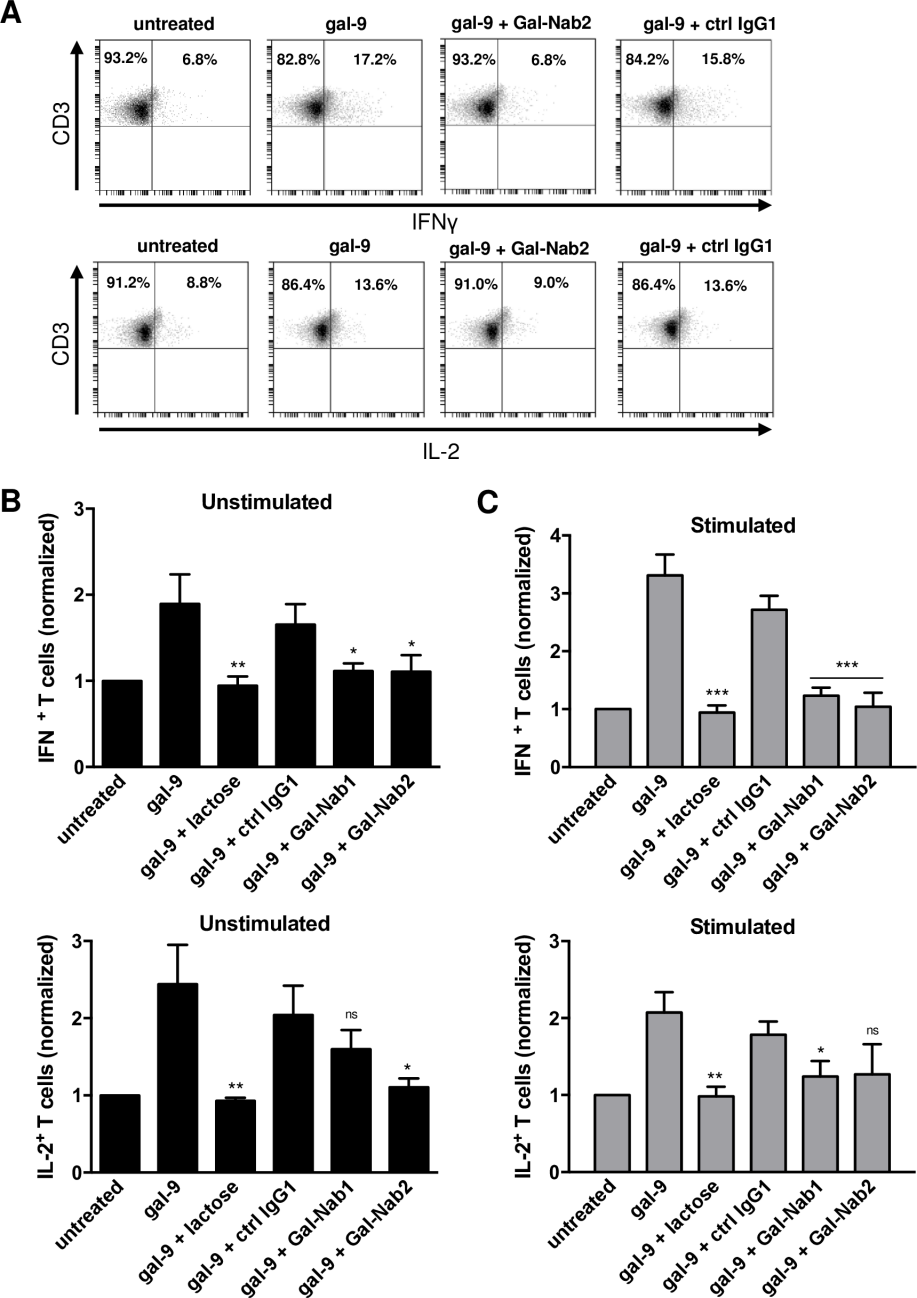
Anti-gal-9 mAbs inhibit the “Th1-like” phenotype shifting induced by gal-9 in resting or stimulated PBMCs. **A-C.** PBMCs from healthy donors were stimulated or not with anti-CD3/CD28 antibodies and treated for one week with human recombinant gal-9 (Gal-9S; 40 nM) with or without combination with lactose (5 mM), control isotype antibody (ctrl IgG1), Gal-Nab1 or Gal-Nab2 (67 nM i.e. 10 μg/mL). Intracellular cytokine expression was assessed by flow cytometry as explained under “Materials and Methods”. At least 50% of the cells were alive. Dead cells were gated out. **A.** Examples of flow cytometry plots obtained with stimulated PBMCs for one donor after gating on the CD3^+^ T cell population: IFN-γ (upper plots) and IL-2 (lower plots) expression were analyzed. **B-C.** Percentages of CD3^+^ IFNγ^+^ (upper histogram) and CD3^+^ IL-2^+^ (lower histogram) cells were normalized with the basal percentages obtained in untreated cells using either unstimulated **(B**) or stimulated **(C)** PBMCs. Data are represented as means ± SEM of four independent experiments with different donors. All statistical differences displayed are compared with gal-9 treatment; ***p<0.001; **p<0.01; *p<0.05; ns: not significant (one-way ANOVA/Dunnet post-test).

**Fig 4:**
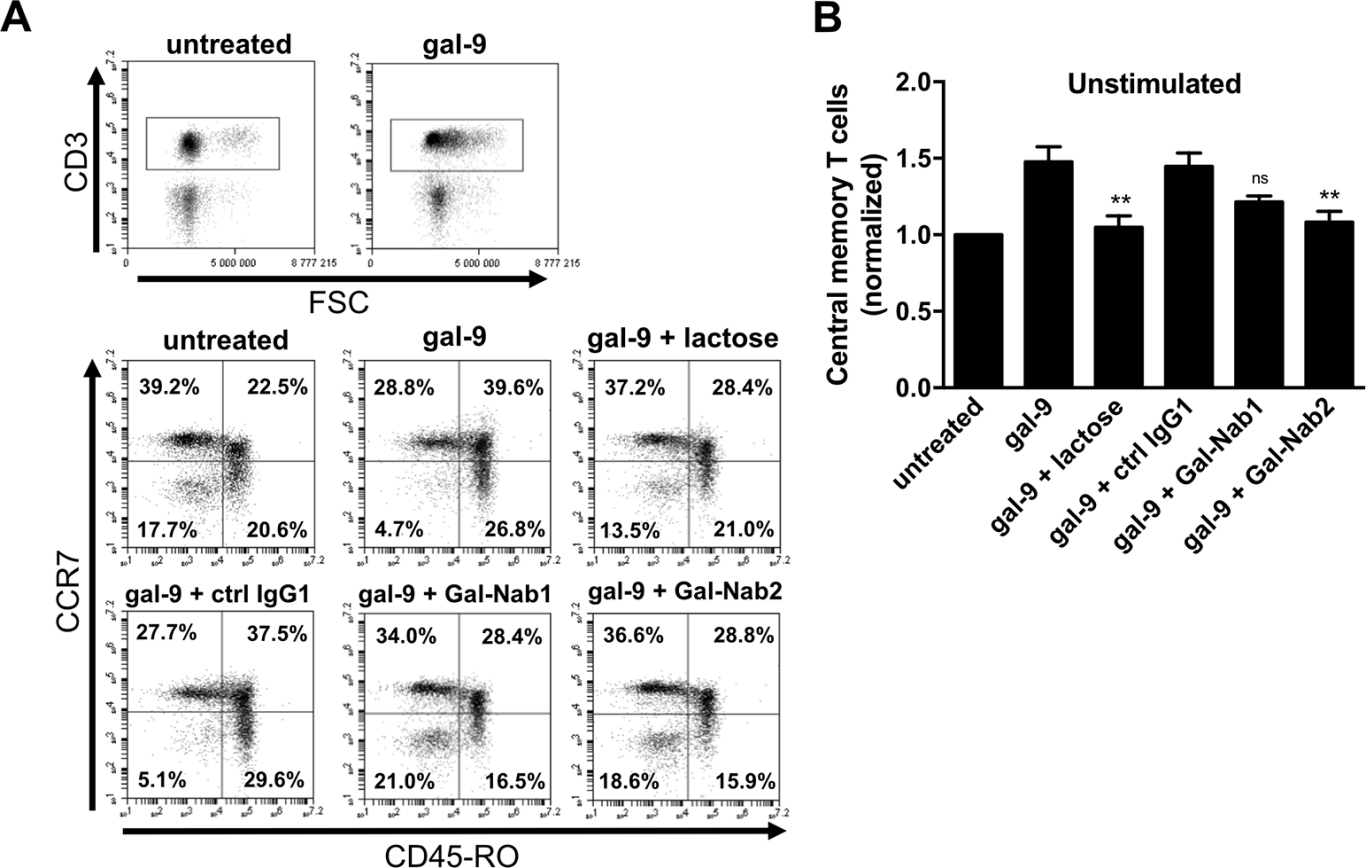
Effect of anti-gal-9 mAbs on the “T_CM_-like” phenotype shifting induced by gal-9 in resting PBMCs. **A-B.** PBMCs freshly isolated from healthy donors were treated for one week with human recombinant gal-9 (Gal-9S; 40 nM) with or without combination with lactose (5 mM), control isotype antibody (ctrl IgG1), Gal-Nab1 or Gal-Nab2 (67 nM i.e. 10 μg/mL). **A.** Flow cytometry analysis was performed to determine the level of naive (CCR7^+^ CD45RO^-^), central memory (CCR7^+^ CD45RO^+^) effector memory (CCR7^-^ CD45RO^+^) and effector (CCR7^-^ CD45RO^-^) T cells within the CD3^+^ population. At least 50% of the cells were alive. Dead cells were gated out. **B.** For each treatment condition, percentages of cells with an apparent central memory phenotype were normalized with the basal level of this subpopulation in untreated cells. Data are represented as means ± SEM of four independent experiments with different donors. **p<0.01; ns: not significant; compared with gal-9 treatment (one-way ANOVA/Dunnet post-test).

It has been shown that, when applied for several days on PBMCs, gal-9 induces the emergence of T cell subpopulations with a Th1-like or memory-like phenotype among the cells surviving gal-9-induced apoptosis [21]. We decided to evaluate the capacity of our mAbs to block these delayed effects of gal-9. Freshly isolated PBMCs (stimulated or not with anti-CD3/CD28 antibodies) were treated for seven days with gal-9 combined or not with lactose or mAbs. On day 7, cells were assessed for intracellular expression of IL-2 and IFN-γ (Fig 3A). We observed that gal-9 increased the proportion of CD3^+^IL-2^+^ and CD3^+^IFN-γ^+^ T cells in unstimulated or stimulated PBMCs, and that this effect was blocked in the presence of lactose. Gal-Nab1 and 2 significantly inhibited the apparent expansion of IFN-γ producing T cells, and to a lesser extent the apparent expansion of IL-2 producing cells (Fig 3B and 3C). At the next stage, we explored the impact of gal-9 and anti-gal-9 antibodies on the distribution of naïve (CCR7^+^ CD45RO^-^), effector memory (T_EM_; CCR7^-^ CD45RO^+^), central memory (T_CM_; CCR7^+^ CD45RO^+^) and effector (CCR7^-^ CD45RO^-^) phenotypes among T cells surviving gal-9 stimulation. Resting PBMCs were treated with gal-9 for one week resulting in a greater proportion of cells with a T_CM_ phenotype. Simultaneously, there was a consistent decrease of the CCR7^-^ CD45RO^-^ CD3^+^ T cells corresponding to effector T cells (Fig 4A). These results confirmed that several days of gal-9 treatment resulted in a shift of T cells towards a T_CM_ phenotype. Moreover, our data indicated that Gal-Nab2 neutralized this shift almost entirely, in the same extent as lactose. Gal-Nab1 tended to inhibit the T_CM_ phenotype shift induced by gal-9, but to a lesser extent without reaching statistical significance (Fig 4B).

### Gal-Nab1 and Gal-Nab2 capture gal-9 with distinct kinetics

We used surface plasmon resonance to analyse the binding characteristics of Gal-Nab1 and Gal-Nab2. In a first series of experiments, recombinant gal-9M was used as the ligand and subjected to the binding of soluble Gal-Nab1 and Gal-Nab2 (Table 1, left column). Reciprocally, in a second phase, Gal-Nab1 and Gal-Nab2 were covalently bound to the chip and soluble recombinant gal-9M was used as the analyte (Table 1, right column). In the first configuration, assessment of binding characteristics is likely to be biased by repetitive binding of the antibodies with a K_D_ reflecting avidity rather than affinity. In this setting, the K_D_ of Gal-Nab1 and Gal-Nab2 were of the same order of magnitude. In the opposite configuration, the K_D_ of Gal-Nab2 (8.79 nM) was almost 10 times smaller than the K_D_ of Gal-Nab1 (88.7 nM), reflecting a lower affinity of Gal-Nab1. The binding speed (k-on) was in the same order of magnitude for both antibodies. However, the speed of elution was much more rapid for Gal-Nab1 than for Gal-Nab2, as shown by the experimental determination of the dissociation and half-life parameters.

**Table 1:** Assessment of the interactions of Gal-Nab1 and Gal-Nab2 mAbs with gal-9M by surface plasmon resonance.

Immobilized ligand	Gal-9M		Gal-Nab1	Gal-Nab2
Analyte	Gal-Nab1	Gal-Nab2	Gal-9M	
**K_D_** (M)	(4.34 ± 1.24) x10^-9^	(1.48x10^-9^ ± 5.30x10^-10^)	(8.87 ± 1.64) x10^-8^	(8.79 ± 5.26) x10^-9^
**K_on_** (s^-1^ M^-1^)	(2.65x10^4^ ± 1.86x10^3^)	(6.11 ± 1.30) x10^4^	(3.74 ± 0.49) x10^5^	(1.90 ± 0.65) x10^5^
**K_off_** (s^-1^)	(1.15x10^-4^ ± 2.87x10^5^)	(9.06 ± 2.61) x10^-5^	(3.32 ± 0.44) x10^-2^	(1.67 ± 0.84) x10^-3^
**t_1/2_** (min)	124.2	127.2	0.35 s	7.32

K_D_, K_on_ and K_off_ were calculated as described under “Material and Methods”.

### Gal-Nab1 and Gal-Nab2 react with nearly identical linear epitopes and share the same heavy chain CDR3 (complementary determining region 3)

In order to better understand the functional differences of these two anti-gal-9 mAbs, we sought to map their epitopes. Using a first set of overlapping peptides, we found that both antibodies were reacting with a segment of gal-9 covering the end of the linker peptide and the beginning of the gal-9 c-terminal CRD (segment spanning aa 202 to 232 in gal-9L; 158–188 in gal-9S). In order to confirm that both antibodies were targeting this segment of gal-9, we used surface plasmon resonance to investigate their binding to an oligopeptide containing aa 208 to 232. This peptide was biotinylated at its c-terminus (hereafter called CTB) and loaded on a streptavidin chip. A biotinylated scramble peptide was used as a control. As seen in Table 2, Gal-Nab1 and Gal-Nab2 were binding the CTB peptide with high avidity whereas no interaction was recorded with the scramble peptide. In addition, we noticed that the apparent affinities of Gal-Nab1 and Gal-Nab2 for the CTB peptide were much greater than their apparent affinities for the intact gal-9M (K_D_ of 1.0x10^-11^ M and 6.05x10^-11^ M, in contrast with 4.34x10^-9^ M and 1.48x10^-9^ M, respectively). Moreover, Gal-Nab1 exhibited a greater affinity than Gal-Nab2 for the CTB peptide whereas it was the opposite for the intact gal-9 (M isoform).

**Table 2:** Assessment of the interactions of Gal-Nab1 and Gal-Nab2 with the CTB oligo-peptide containing aa 208 to 232 of gal-9 by surface plasmon resonance.

Immobilized ligand	Scramble peptide	CTB-gal-9 peptide	Scramble peptide	CTB-gal-9 peptide
Analyte	Gal-Nab1		Gal-Nab2	
**K_D_** (M)	No binding	(1.0 ± 0.48) x10^-11^	No binding	(6.05 ± 3.92) x10^-11^
**K_on_** (s^-1^ M^-1^)		(2.3 ± 0.63) x10^6^		(5.45 ± 0.95) x10^5^
**K_off_** (s^-1^)		(2.3 ± 0.28) x10^-5^		(3.3 ± 1.41) x10^-5^
**t_1/2_** (min)		8.37		6.23

K_D_, K_on_ and K_off_ were calculated as described under “Material and Methods”.

**Fig 5:**
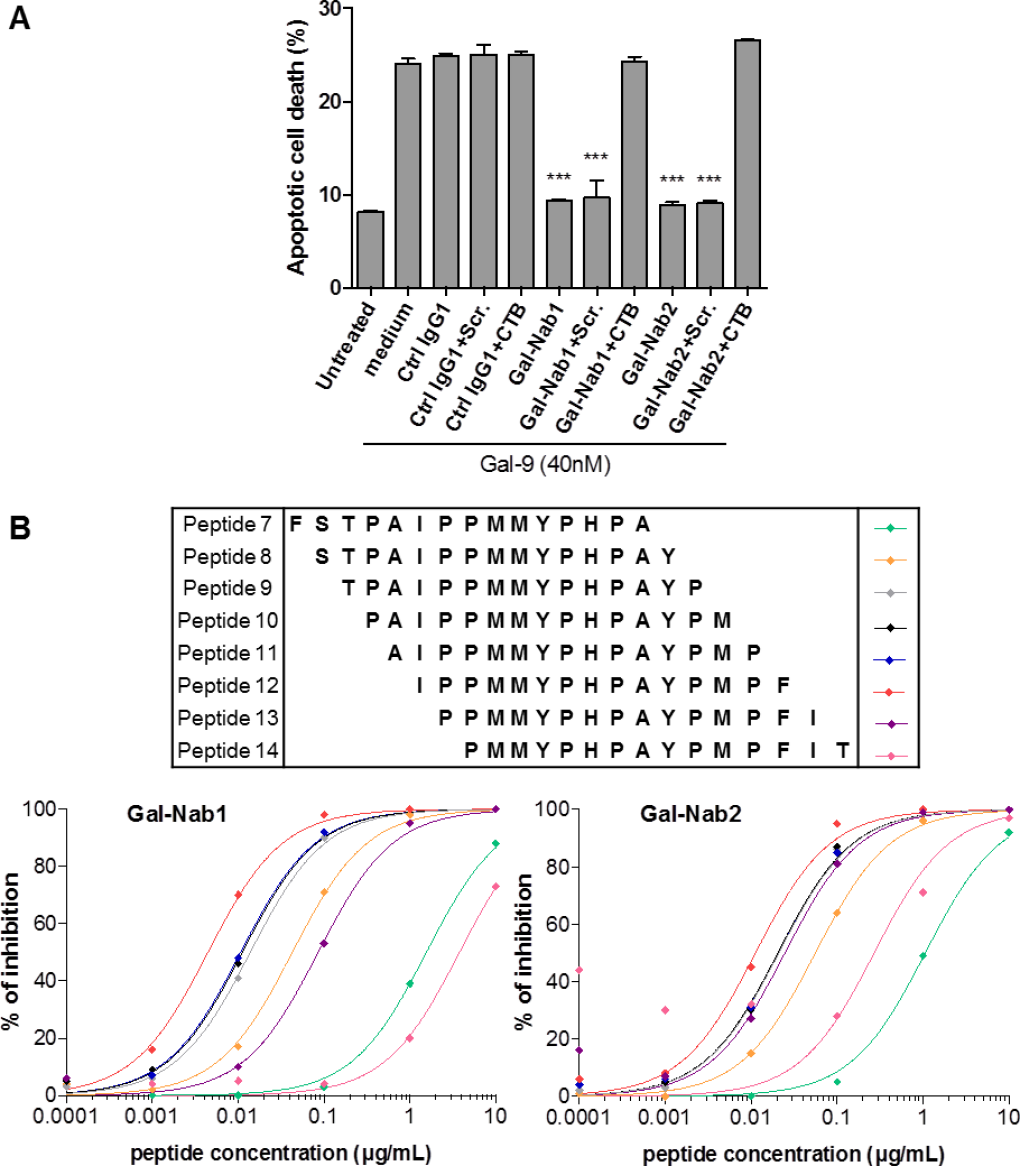
Gal-9 epitope recognition by Gal-Nab1 and Gal-Nab2. **A.** Apoptotic cell death assessed by flow cytometry in CD3^+^ T cells treated for 36h with gal-9 (gal-9S; 40 nM) alone or in combination with mAbs (ctrl IgG1, Gal-Nab1, Gal-Nab2; 67 nM) pre-incubated or not with scramble peptide (Scr.) or gal-9 CTB-peptide (6.7 μM). **B.** Human recombinant gal-9 was immobilized in 96-wells plates and binding of Gal-Nab1 and Gal-Nab2 mAbs was measured after pre-incubation with overlapping peptides representative of human gal-9, as described under “Material and Methods”. Gal-Nab1 **(left)** or Gal-Nab2 **(right)** were then detected using secondary HRP-conjugated anti-mouse antibodies. Percentages of inhibition induced by each peptide were calculated from the absorbance data as described under “Material and Methods”.

We also confirmed the specific binding of this peptide to our mAbs by performing a functional assay (Fig 5A). After pre-incubation with the scramble peptide, Gal-Nab1 and Gal-Nab2 inhibit gal-9-induced apoptosis of CD3^+^ primary T cells. However, pre-incubation of the mAbs with the CTB-peptide completely abolishes their neutralizing capacity confirming their high affinity for an epitope carried by this peptide.

Finally, in a second round of epitope mapping, we used a set of 15 overlapping 15-mers spanning aa 202–230 with a shift of only one aa between consecutive oligo-peptides. We assessed the capacity of these peptides to block the interaction between Gal-Nab1/Gal-Nab2 and gal-9. As shown in Fig 5B, the peptide 12 induces the most potent inhibition for both antibodies. The peptide 13 had almost the same impact as the peptide 12 for Gal-Nab1 but not for Gal-Nab2, underlying the contribution of isoleucine 213 for Gal-Nab1 binding to gal-9 in contrast to Gal-Nab2. Taking in account the impact of the different peptides, we could determine the sequences of the linear epitopes recognized by our mAbs: “IPPMMYPHPAYP” (aa 213 to 224 in gal-9L; 169–180 in gal-9S) for Gal-Nab1 and “PPMMYPHPAYP” (aa 214 to 224 in gal-9L; 170–180 in gal-9S) for Gal-Nab2. These data mean that both mAbs react with the same linear epitope, at the exception of one residue (I; isoleucine) which is more critical for Gal-Nab1 binding to gal-9. In order to get some insights on structural similarities and differences between Gal-Nab1 and Gal-Nab2, we sequenced the variable portions of their heavy and light chain genes. As shown in Table 3, the CDR3 of their heavy chain were identical. Only minor differences were recorded for the CDR1 of the heavy chains and the CDR2 and 3 of the light chains. Greater variations were observed in the CDR2 of the heavy chains and CDR3 of the light chains.

**Table 3:** Sequences of heavy and light chains CDRs for Gal-Nab1 and Gal-Nab2.

	Heavy chain			Light chain (κ)		
	CDR1	CDR2	CDR3	CDR1	CDR2	CDR3
**Gal-Nab1**	GYTFT**D**YTIH	WFYPGS**H**S**IK**YNE**Q**F**K**D	HGGYDGFDY	KSSQSL**F**YS**T**NQKNYLA	WASTR**E**S	QQYY**YF**P**Y**T
**Gal-Nab2**	GYTFT**E**YTIH	WFYPGS**G**S**ME**YNE**K**FD	HGGYDGFDY	KSSQSL**L**YS**N**NQKNYLA	WASTR**G**S	QQYY**SY**P**F**T

Differences in amino acids between the antibodies sequences are marked in bold characters.

### Gal-Nab1 and Gal-Nab2 cross-react with murine gal-9

**Fig 6:**
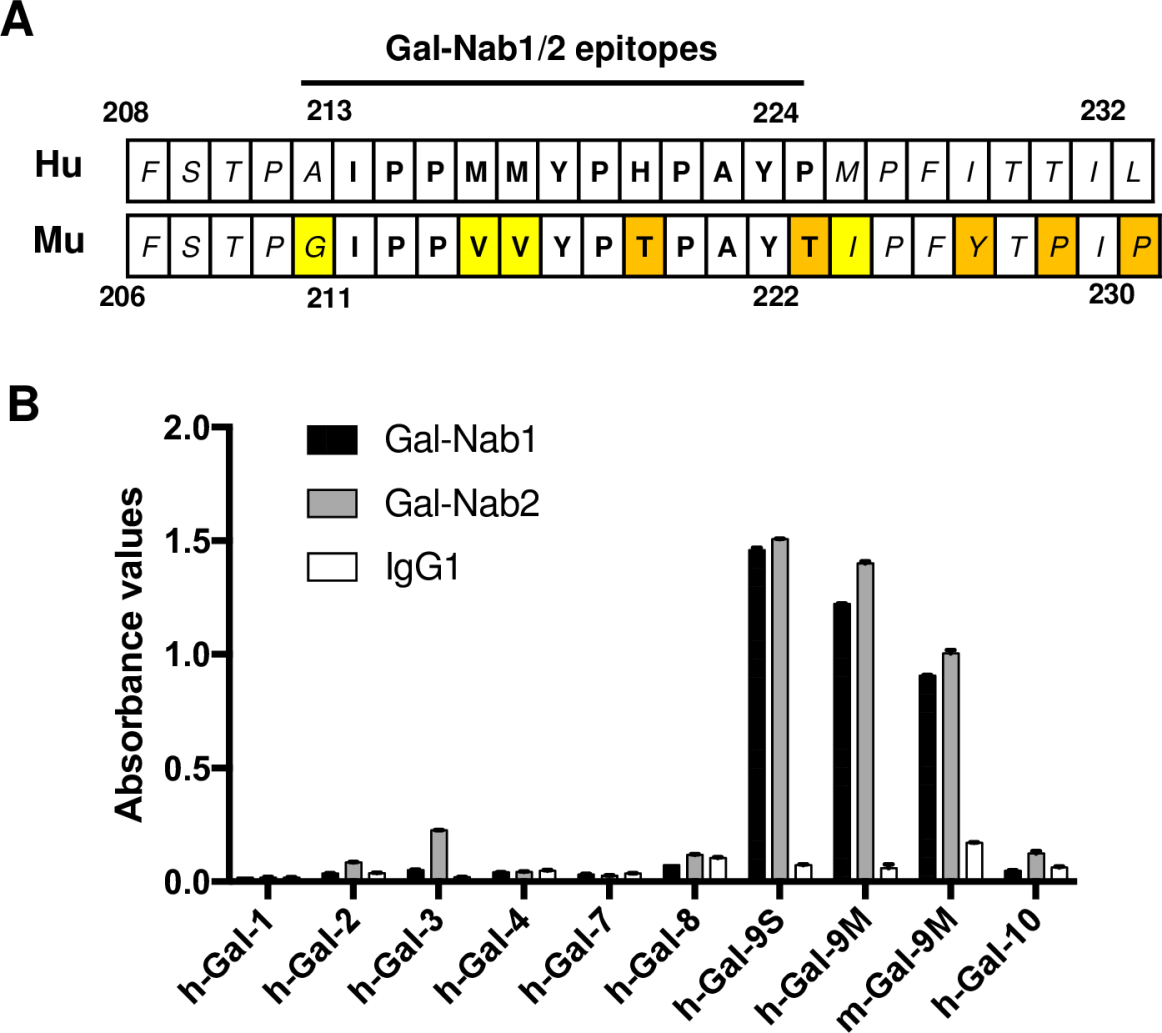
Partial cross-reaction of Gal-Nab1 and Gal-Nab2 with recombinant murine gal-9. **A**. Comparative display of the 208–232 aa sequence of human gal-9 (upper line) and its homologous aa sequence in murine gal-9 (lower line) (aa 206–230 for the long isoform, based on the UniProtKB database http://www.uniprot.org/uniprot/O00182 and O08573). Variant aa are highlighted in yellow (conservative changes) or in orange (non-conservative changes). Within the epitopes of Gal-Nab1 and Gal-Nab2, there are only 2 non-conservative changes. **B**. The binding of Gal-Nab1 and Gal-Nab2 to recombinant galectins was assessed by direct ELISA performed in triplicate. Target proteins were human galectins 1 to 4, 7, 8 and 10 (h-Gal); the S and M isoforms of human gal-9 (h-Gal-9S and M) and the M isoform of murine gal-9 (m-Gal-9M).

**Fig 7:**
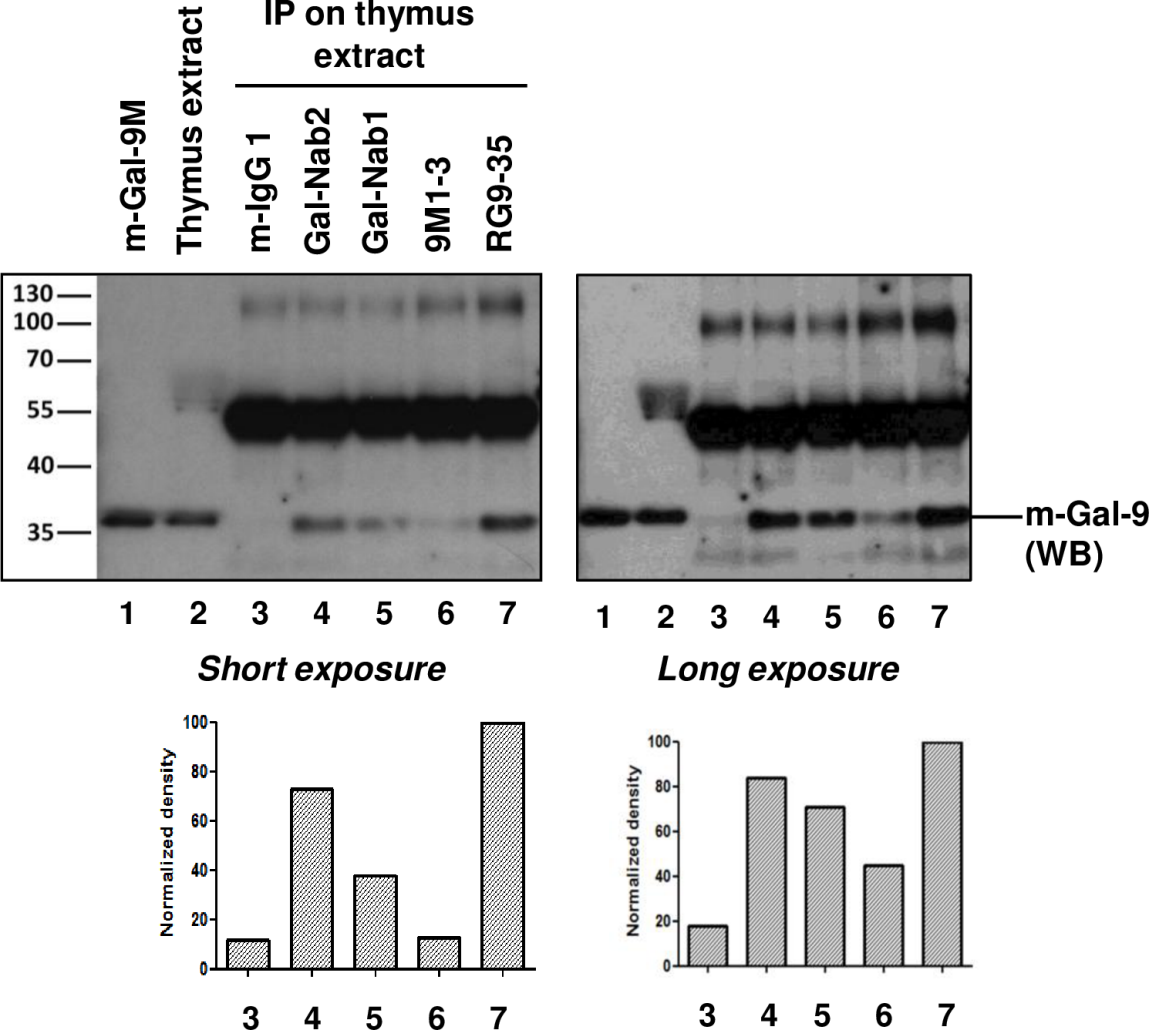
Partial cross-reaction of Gal-Nab1 and Gal-Nab2 with native murine gal-9. Western blot (WB) analysis of cold immuno-precipitation (IP) products obtained with various anti-gal-9 antibodies. The source of native gal-9 was a protein extract from a murine thymus. Concomitant IP were performed with non-specific mouse IgG1 (negative control), Gal-Nab1 and Gal-Nab2, 9M1 (a commercial antibody directed to human gal-9) and RG9-35 (a commercial antibody directed to murine gal-9). 15 ng of recombinant murine gal-9 M (lane 1) and 25 μg of crude murine thymus extract (lane 2) were used as WB controls. The primary antibody used for WB detection of murine gal-9 was a polyclonal goat serum. The secondary antibody reacted with this primary but also with the immunoglobulin heavy chains eluted from the beads giving strong bands at a molecular weight of about 55 Kd. These bands were of similar intensities for all five IP conditions, suggesting that bead capture of the immune complexes was in the same range of efficiency. The bands resulting from gal-9 staining (36 Kd) were assessed by densitometry (graphs for long and short exposure are presented at the bottom of the figure).

Because investigations in murine models are generally regarded as a pre-requisite for development of therapeutic antibodies, we investigated the capacity of Gal-Nab1 and 2 to cross-react with recombinant and native murine gal-9. First, the aa sequence containing the Gal-Nab1 and 2 overlapping epitopes (aa 213–224 of human gal-9) was compared to the homologous segment of murine gal-9 (aa 211–222 of murine gal-9) (Fig 6A). Out of 12 aa, four are divergent with only two non-conservative changes (aa 218 and 222 of murine gal-9). These observations were in favor of a cross-reactivity of Gal-Nab1 and 2 with murine gal-9. As shown in Fig 6B, Gal-Nab1 and Gal-Nab2 did cross-react with recombinant murine gal-9M as evaluated by ELISA. In contrast, there was no cross-reactivity with human galectins distinct from human gal-9 except a marginal reaction with human galectin-3. In order to explore the binding of Gal-Nab1 and Gal-Nab2 to native murine gal-9, we performed a cold immunoprecipitation assay using a protein extract from murine thymus as the source of native gal-9. The precipitation output of Gal-Nab1 and 2 was compared to the output of two other monoclonal antibodies, 9M1-3 and RG9-35, designed to react with human and murine gal-9, respectively. As shown in Fig 7, Gal-Nab2 and, to a lesser extent, Gal-Nab1, were able to precipitate a substantial amount of murine gal-9. As expected, the recovery of native murine gal-9 was maximal with RG9-35. It was very low with 9M1-3. Based on these data, one may consider a direct exploration of the therapeutic potential of Gal-Nab1 and 2 in murine syngeneic models.

## Discussion

Hijacking physiological mechanisms involved in the down-regulation of the immune responses is one major component in the expansion strategy of malignant cells and pathogenic viruses. Conversely, blocking mechanisms of immune tolerance towards tumor cells or viral pathogens increasingly appears as an essential–if not mandatory–component of the treatment of most malignancies and viral diseases [40, 41]. The benefit of this strategy is remarkably illustrated by the success of several checkpoint inhibitors like Ipilimumab or Nivolumab in various types of malignancies [42, 43]. So far gal-9 has been neglected as a potential therapeutic target although it is strongly suspected to impair the immune response in various pathological conditions, especially in nasopharyngeal, pancreatic, and renal carcinomas as well as chronic viral hepatitis [24, 26–28, 30, 44]. In addition, most authors seeking therapeutic inhibition of other galectins, for example galectin-1 or -3, have mainly resorted to small molecules especially glycomimetics [45, 46]. However, neutralizing antibodies are expected to carry several benefits over small molecules: 1) a greater specificity for each type of galectin species; 2) inhibitory effects restricted to extra-cellular galectins; 3) extensive knowledge already available on pharmacokinetics/pharmacodynamics and bio-distribution; 4) a known regulatory landscape. Nevertheless, so far, to our knowledge, there was no report on antibodies neutralizing gal-9 or other galectins produced with a therapeutic intent [45]. In this context, our general objective was to pave the way for the development of humanized therapeutic antibodies neutralizing the unwanted immunosuppressive effects of gal-9.

We have obtained two lead monoclonal antibodies which neutralize gal-9 in various biological assays performed *in vitro*. They have many convergent effects. Their most promising common effect with regard of therapeutic development is their protection against the induction of the apoptosis of conventional primary T-cells by exogenous gal-9. Later on, additional investigations will be necessary to elucidate the mechanisms underlying this neutralizing activity. It is noteworthy that neither Gal-Nab1 nor Gal-Nab2 binds to T cells (S2 Fig). They might prevent the interaction of gal-9 with some of its receptors on the plasma membrane of the target cells by steric hindrance, modification of gal-9 3D conformation or impairment of its dimerization [47]. In other types of assays—cellular or a-cellular—Gal-Nab1 and Gal-Nab2 exhibit distinct functional properties, although they share the same heavy chain CDR3 and react with nearly the same linear epitope. The main differences are the rapid dissociation of Gal-Nab1 from gal-9 and its own stimulating effects on non-lethal PS translocation and calcium mobilization induced by extra-cellular gal-9 in Jurkat cells. One hypothesis is that the differences recorded in the CDR-1 and -2 of Gal-Nab1 and Gal-Nab2 determine distinct types of paratope/epitope interfaces. In addition to the 213–224 aa segment of gal-9 included in the CTB peptide, Gal-Nab1 and 2 are likely to react with other parts of the protein. Their apparent greater affinity for the CTB peptide than for the whole protein suggests that the other contact points of the three-dimensional epitopes have negative contributions to the stability of the antigen/antibody complexes. These negative contributions seem to be greater for Gal-Nab1 than for Gal-Nab2. More investigations will be required to address this hypothesis. Another intriguing observation is that Gal-Nab1 does not modulate in the same way the responses of Jurkat cells and primary T cells stimulated by extra-cellular gal-9. To address this question, one needs to remember that Jurkat cells are malignant cells and therefore have a glycan profile on their plasma membrane which is not identical to primary CD3^+^ cells [48]. This might determine specific modalities of gal-9 interactions with Jurkat cells.

None of our two lead antibodies cross-react with human galectins 1 to 4, 7, 8 and 10 as shown in Fig 6B. In contrast, they show partial cross reaction with recombinant and native murine gal-9 which is consistent with the fact that the murine peptide sequence homologous to their target epitopes is highly conserved in rodents. Since it is even more conserved in primates, we can anticipate a possible use of these antibodies for preclinical evaluation in primates.

Further experiments are warranted to analyse more specifically the impact of gal-9 and our mAbs on various T-cell subpopulations. This is particularly important since gal-9 has been shown to be important for Tregs expansion and activity [6, 10, 49]. Thus, anti-gal-9 mAbs may limit the immunosuppressive activity of Tregs and therefore improve the overall anti-tumor immune response. Results presented in Figs 3 and 4 confirm previous data from Gooden *et al* who showed that following the initial wave of apoptosis, gal-9 treatment of PBMCs results in the emergence of T cells with a phenotype suggestive of Th1 cells (CD3^+^IFN-γ^+^) and central memory T cells (CD45RO^+^ CCR7^+^) [21]. We found that Gal-Nab1 and 2 prevent the late emergence of these T cell subpopulations. These observations raise the possibility that the expected benefits of anti-gal-9 antibodies might be balanced by some unwanted immunosuppressive effects. However, this possibility might not be true, and that for several reasons. First, we need further evidence that the increase in the percentages of CD3^+^IFN-γ^+^ and CD45RO^+^ CCR7^+^ cells following the apoptotic wave is real and not just a consequence of the depletion of other subpopulations. Proliferation assays will be required to address this point. Next, in light of several recent publications that demonstrate dissociation between the function of some T cells and their membrane phenotype, one cannot exclude that these populations have in fact suppressive functions [50–52]. Sorting of these cells and direct evaluation of their potential suppressive activity will be required to answer this question. Finally, antigen-independent activation of conventional T cells by gal-9 might not be beneficial: it might favor permanent inflammation in the tumor microenvironment without enough focus of T cell cytotoxicity against malignant cells or virus-infected cells. For all these reasons, we believe that most effects of our mAbs are likely to contribute to an immune restoration in pathological conditions with excessive gal-9 production. These remarks underline the importance of future investigations in pre-clinical models, especially murine models. Our data demonstrate that Gal-Nab1 and 2 partially cross-react with murine gal-9. Therefore they will be probably well suited for use in syngeneic murine tumor models in order to assess their overall impact on host-tumor relationships.

## Supporting information

S1 Fig Capture of biotinylated galectins on surface-bound antibodies. The binding of Gal-Nab1, Gal-Nab2 and control mouse IgG1 antibodies to gal-9 was assessed by direct ELISA as described under “Materials & Methods” with the difference that biotinylated gal-9S (1 nM) was incubated during 1h at 37°C in antibody-coated wells in the presence or absence of lactose (20mM) before washing and revelation.
(TIF)

S2 Fig Gal-9 surface staining on primary T cells. CD3^+^ purified T cells were stimulated (CD3/CD28 coated beads) or not and incubated with anti-MHC-I antibodies (as positive control), anti-gal-9 mAbs (9M1, Gal-Nab1 and Gal-Nab2; 5 μg/ml) or mouse irrelevant IgG1 for 30 min at 4°C. Cells were then washed in ice-cold PBS and incubated 30 min at 4°C with rat anti-mouse antibodies (Biolegend) coupled to fluorescein isothiocyanate (FITC). Grey histograms represent the background of fluorescence obtained with the irrelevant IgG1 as primary antibodies.
(TIF)
