# Supplementary figures and images for: Characterization of neutralizing antibodies reacting with the 213-224 amino-acid segment of human galectin-9
Source: PLoS One. 2018 Sep 11;13(9):e0202512. doi: 10.1371/journal.pone.0202512 (PMC6133441; doi:10.1371/journal.pone.0202512)

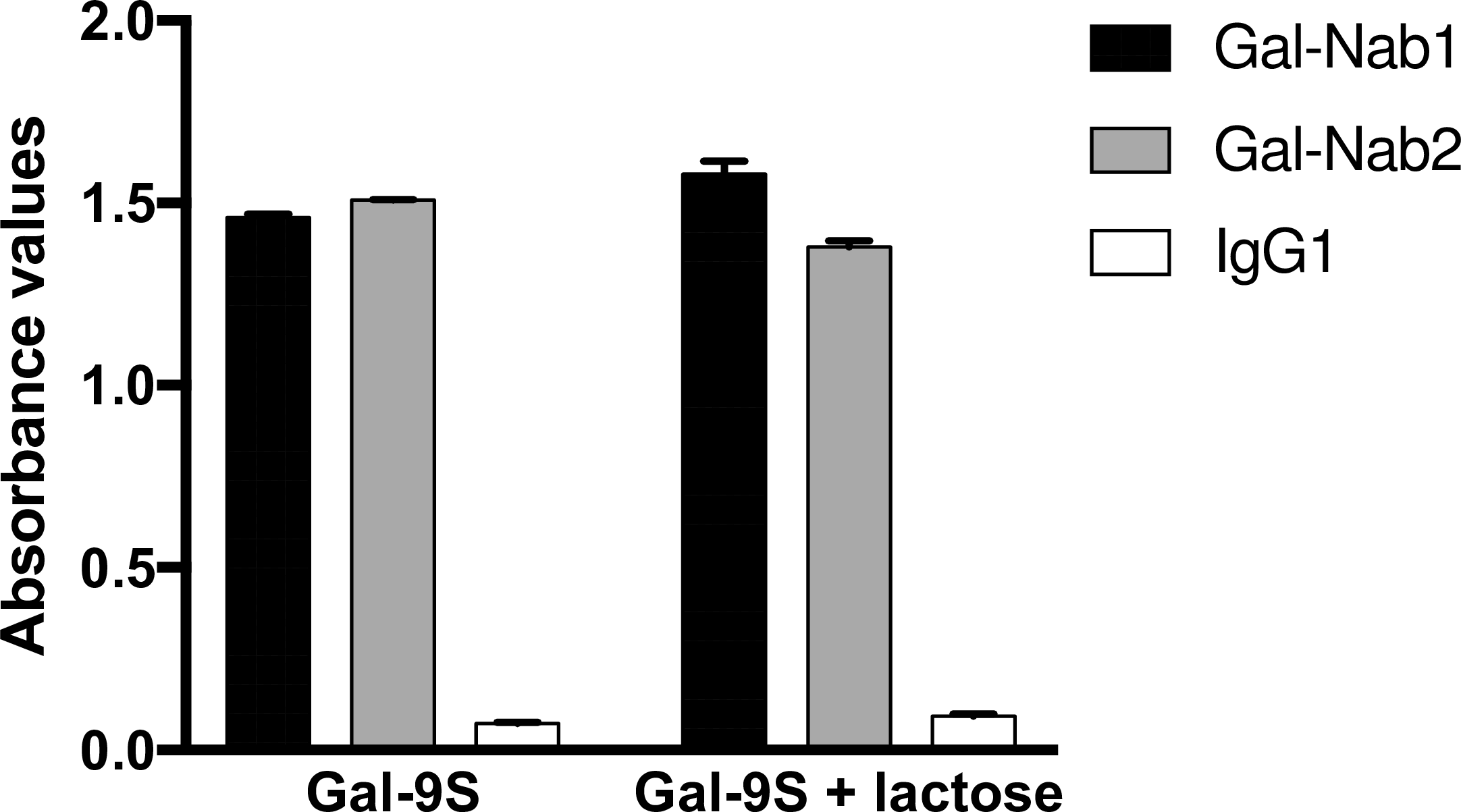

Supplement: S1 Fig — The binding of Gal-Nab1, Gal-Nab2 and control mouse IgG1 antibodies to gal-9 was assessed by direct ELISA as described under “Materials & Methods” with the difference that biotinylated gal-9S (1 nM) was incubated during 1h at 37°C in antibody-coated wells in the presence or absence of lactose (20mM) before washing and revelation. (TIF) [file pone.0202512.s001.tif]

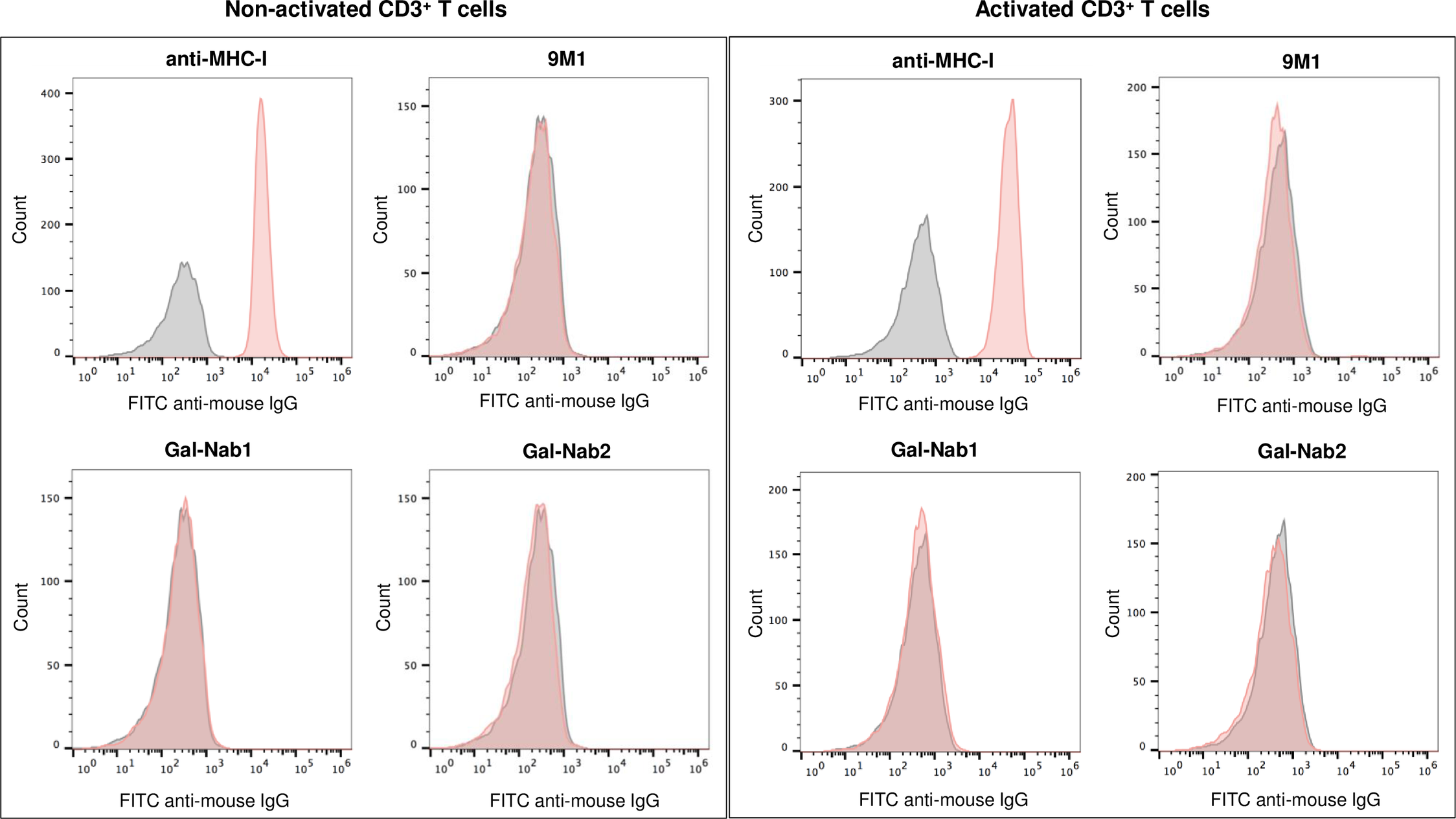

Supplement: S2 Fig — CD3+ purified T cells were stimulated (CD3/CD28 coated beads) or not and incubated with anti-MHC-I antibodies (as positive control), anti-gal-9 mAbs (9M1, Gal-Nab1 and Gal-Nab2; 5 μg/ml) or mouse irrelevant IgG1 for 30 min at 4°C. Cells were then washed in ice-cold PBS and incubated 30 min at 4°C with rat anti-mouse antibodies (Biolegend) coupled to fluorescein isothiocyanate (FITC). Grey histograms represent the background of fluorescence obtained with the irrelevant IgG1 as primary antibodies. (TIF) [file pone.0202512.s002.tif]
